# Zhuang-Mandarin bilingual children in rural China and the role of grandparental input in early bilingualism

**DOI:** 10.1371/journal.pone.0326671

**Published:** 2025-06-26

**Authors:** Ziyin Mai, Patrick C.M. Wong, Stephen Matthews, Virginia Yip, Hanbo Liao, Jiaqi Nie, Yue Chen

**Affiliations:** 1 Department of Linguistics and Modern Languages, Chinese University of Hong Kong, Shatin, Hong Kong; 2 Department of Linguistics, School of Humanities, University of Hong Kong, Pokfulam, Hong Kong; University of Education, PAKISTAN

## Abstract

Linguistic properties of bilingual input and their relations with acquisition outcomes are being intensively studied in current research on early bilingual development. Motivated by emerging interests in grandparental input and the unique language profile of Zhuang-Mandarin bilinguals in rural China, this article reports an exploratory study investigating bilingual input-outcome relations in two groups of age-matched kindergarteners who were primarily cared for by Zhuang-speaking grandmothers (GRA group, n = 4) and by Zhuang-Mandarin bilingual mothers (MOT group, n = 5) respectively. Through (grand)parental questionnaires, caregiver-child interaction recordings and direct assessments of the children, we collected two waves of data around the beginning and the end of Mandarin-medium kindergarten, focusing on the input and the outcomes respectively (Time 1/Time 2 design). Our findings show that at both times, the grandparents spoke considerably larger proportions of Zhuang to the children than the mothers, who had completely shifted to Mandarin by Time 2. Both groups of children were dominant in Mandarin at Time 2, demonstrating quantitatively and qualitatively similar production performance, but only the GRA children were able to produce words and narratives in Zhuang. It is argued that early sequential bilingualism actively promoting and supporting grandparental input in Zhuang in addition to school input in Mandarin is beneficial to the preservation of Zhuang as a minority language and mastery of the national majority language. Implications for language intervention and planning concerning minority languages in rural China are discussed.

## Introduction

Bilingualism is a wide spectrum shaped by a complex set of interactional experiences at interpersonal, familial and sociocultural levels [1]. Young children raised by caregivers who regularly use two languages with them appear to acquire two languages naturally and seemingly effortlessly. In recent decades, the quantity and quality of home language input and their relations with bilingual acquisition outcomes in the early years have been intensively investigated across bilingual populations, yielding rich findings centered on maternal input in heritage/immigrant language versus majority language contexts. Despite sporadic evidence suggesting a positive role of grandparents in the maintenance of the minority language (e.g., [2–4]), little research has directly examined the language input provided by grandparents and its role in long-term development of bilingual children.

Early Zhuang-Mandarin bilingualism in China presents an interesting case to explore grandparental input. Zhuang is a “major” minority language spoken by approximately 20 million speakers in Guangxi, the majority of them proficient in both Zhuang and Mandarin [5, 6]. Recent years have seen a decline in the number of Zhuang speakers, giving rise to the speculation that Zhuang is becoming an endangered language, reflecting socio-economic and policy-related factors associated with urbanization in rural China [7]. According to the Language Endangerment Index [8], the crucial factor determining language endangerment is intergenerational transmission, that is, the degree to which the language is being passed on to the youngest generation at the family level. Given this, it is essential to observe how adult native speakers of Zhuang use the language with the children that they provide care for.

This study investigates the role of grandparental input in early bilingual development in Zhuang and Mandarin by collecting input and outcome data from two groups of children raised by Zhuang-speaking grandmothers in rural villages (n = 4) and Zhuang-Mandarin bilingual mothers in urban areas (n = 5) respectively at two time points, at the beginning and the end of Mandarin-medium kindergarten. Through this small-scale and exploratory study, we aim to provide the first linguistic sketch of grandparental input in ethnic minority households in China, paving the way for larger-scale documentation and interventions. Below we provide a linguistic overview of the Zhuang language and its speakers, followed by a review of studies on home language input in early bilingual development, before presenting the methods and findings of our own study.

### Zhuang as an ethnic minority language in China

Standard Mandarin Chinese (Putonghua) is the national language of China and the lingua franca across different regions of the country, as well as the medium of instruction in mainstream primary and secondary education [6]. In addition to Mandarin and many regional varieties of Mandarin (“dialects”), more than 120 languages are spoken by over 55 ethnic minority groups in China, accounting for 8.49% of the total population [7, 9]. Zhuang is a tonal language belonging to the Tai-Kadai language family [10]. As the largest minority language in China, Zhuang has 17-20 million speakers, most of whom reside in the rural area of Guangxi Zhuang Autonomous Region (hereafter “Guangxi”, [5, 7]). Linguistic analyses recognize two dialect groups of Zhuang: the northern group and the southern group. The Standard Zhuang dialect is based on a northern dialect spoken in the Shuangqiao Township of Wuming County in Guangxi, with a standard Romanised orthography.

Although the core lexicon of Zhuang has a different origin from that of Mandarin, all Zhuang dialects have numerous loanwords from Chinese languages in different historical layers. For example, a whole set of numerals is borrowed from Old Chinese and is thus very close to those of southern Chinese dialects such as Cantonese and Hakka [10]. Based on the description in Luo [11], the grammar of standard Zhuang (as well as many well-documented varieties) shares many similarities with Mandarin Chinese. To list a few, both languages prefer subject-verb-object (SVO) as the canonical word order, with frequently attested sentential topics and null arguments conditioned by discourse; questions are formed with the *wh*-elements staying in situ or through the A-not-A form; the pronoun system is simple and unmarked for gender; verbs are marked with aspect (rather than tense) markers and can be followed or preceded by prepositional and adverbial phrases, subject to event semantic conditions that are similar to their Mandarin counterparts; reduplication of verbs, adjectives, nouns and classifiers are attested; both have a passive construction carrying an adversative meaning (“to suffer/endure”). A notable difference is that, relative clauses in Zhuang are post-nominal, whereas those in Mandarin are pre-nominal, deviating from most SVO languages.

Children who develop Zhuang as their first language (L1) in Guangxi also acquire Mandarin, either because Mandarin is another language spoken in the family or because it is taught at kindergarten and used in the larger community. In either case, Zhuang-L1 children in Guangxi develop bilingual proficiency very early. Whilst Zhuang is the native language of the Zhuang population, speakers of Zhuang have become increasingly bilingual in Mandarin or other dialects of Chinese in the past few decades (reaching around 70% by the early 2000s, [6]). Despite national and regional policies to preserve and protect Zhuang, the number of Zhuang speakers among younger generations reared in the urban areas of Guangxi has been declining, and Mandarin is predominantly adopted as the language for communication among young Zhuang-Mandarin bilinguals. Yet research on the acquisition of Zhuang, whether monolingually or bilingually alongside Mandarin, by young children in Guangxi remains scarce.

### Early childhood bilingualism and home language input

Research on childhood bilingualism involving a heritage/minority language and a societal majority/dominant language has shown that the minority language in these cases develops quite differently from the way it develops in a monolingual society where the language is dominant [12–14]. For example, at the lexical level, young heritage speakers of Persian in New Zealand (age 6-18) had limited knowledge of productive and receptive vocabulary compared to matched monolinguals, and the vocabulary size of the heritage language correlated negatively with the age when the heritage children were exposed to the majority language environment [15]. At the grammatical level, school-age heritage speakers of Mandarin in the UK exhibited protracted development in various aspects of prominent grammatical constructions including the *ba*-construction, resultative verb compound and placement of prepositional phrases in Mandarin, which was related to the amount and quality of Mandarin input at home [16–18].

Researchers broadly agree that reduced input and use in the heritage L1 at the school age is one of the most important factors underlying the differences between heritage and monolingual development. Studies have provided robust evidence that across families, the number of parents who address the children in the heritage L1, the use of the heritage language with sibling(s), and activities conducted in the heritage L1 (play, media, etc.) all contribute to the maintenance (or loss) of the heritage L1 at school age ( [19, 20], etc.). However, little has been done to understand the linguistic properties of the language input provided by grandparents and its role in early bilingual development in a minority-L1 setting. Xiang and Makarova [21] interviewed 60 school-age heritage Chinese children in Canada (age 6-13) on their language activities and assessed their proficiency in Mandarin. Close to half of the households had grandparents living with the children, and among them, around 33% were identified by the children as the main source of Mandarin input. In addition, children with co-living grandparents outperformed those without them in terms of grammatical accuracy in their production. Li and Matthews [2] reported a longitudinal case study in which first-generation immigrant grandparents in the US passed on the heritage language (Mandarin) to their third-generation grandchildren through regular daily interaction with them and motivated second-generation parents to re-learn their heritage language. Overall, emerging linguistic and sociolinguistic evidence suggests that grandparental input and grandparent-child interaction play an important role in heritage language maintenance, which partially motivates our study.

The heritage L1 is not the only language in the combination that is affected by the input factor. Acquisition of the majority language (usually the second language (L2) of the heritage children) is also sensitive to various aspects of the input, especially its timing and setting. Research on child L2 acquisition has examined the input and environmental factors across L1-L2 pairings and acquisition settings. Robust evidence has shown that native-likeness in the L2 is strongly associated with early onset of L2 acquisition (e.g., [22]), although an early onset does not guarantee native-like performance across linguistic domains and only explains a portion of attainment in the L2 [23]. For immigrant children who speak a heritage L1 at home and are immersed in the majority L2 at school, perception and production of L2 phonemes seem to be facilitated by L1-L2 similarities and lags behind in L2-specific sounds at the early stages, but preschool children are able to catch up and match their monolingual peers, usually within one year of systematic L2 exposure [24, 25]. Lexical development also seems to be highly responsive to L2 immersion through schooling, with preschoolers meeting native-speaker expectations in vocabulary tests after 2-3 years of exposure to the majority L2 (e.g., [26]). Grammatical development, however, yields a less clear picture. Morphological agreement and inflection are especially problematic, causing persistent production errors in child L2 learners even after six years of immersive learning of the L2 (e.g., [27]). Importantly, when both the L1 and the L2 regularly instantiate morphological inflection, accuracy in L1 inflection is a better predictor of accuracy in L2 inflection than age of onset and amount of exposure of the L2, pointing to positive L1 influence (e.g., [28]). However, less is known about L1-L2 pairs of isolating languages that do not have rich morphological marking. Given the similarities between the Zhuang and Mandarin grammatical systems, it is likely that sequential Zhuang-Mandarin children can catch up with Mandarin-L1 children or simultaneous Zhuang-Mandarin children quite rapidly. Our study tests this hypothesis.

## Current study

### Research questions and predictions

Our review above has revealed the paucity of research on the role of grandparental input in children’s acquisition of L1 as a heritage/minority language in the presence of a societal dominant language, as well as on the potential facilitative effects of L1 knowledge in acquiring a similar L2, which may mitigate the disadvantage of later onset age of the societal majority language. Early Zhuang-Mandarin bilingualism in Guangxi is an ideal testbed to address both issues. Guangxi is among the labour-exporting provinces in China from which young workers migrate to other cities for better employment opportunities, leaving young children to be cared for by their grandparents [29]. Additionally, given that Zhuang and Mandarin display similarities and close correspondences in many grammatical structures, cross-linguistic influence between Zhuang and Mandarin should be largely positive. Against this backdrop, our study investigates the development of Zhuang and Mandarin in two groups of bilingual preschoolers in Debao County in western Guangxi, one of them receiving input primarily from Zhuang-speaking grandparents and the other from bilingual mothers. We asked the following research questions:

1. How does the language input provided by Zhuang-speaking grandparents in rural villages compare with that provided by Zhuang-Mandarin bilingual mothers in urban town areas in terms of language choice and quality?
2. How do the children who were primarily cared for by the grandparents compare with those raised by the mothers in developing bilingual proficiency in Zhuang and Mandarin at kindergarten age?

Given the inter-generational changes in Zhuang-Mandarin bilingualism in Guangxi reviewed above, we predict that grandparents should address their grandchildren more often in Zhuang and the mothers more often in Mandarin. For bilingual development at kindergarten age, we predict that children who are cared for by their grandparents and have thus received rich and consistent input in Zhuang will outperform children raised by mothers who address their children in Zhuang less often in terms of Zhuang proficiency. Meanwhile, due to the close linguistic affinities between Zhuang and Mandarin and the immersive environment of Mandarin at kindergarten, we expect that the two groups of children will reach similar proficiency in Mandarin by this age.

### Participants and the Time 1/Time 2 design

Four bilingual preschoolers (JD, JR, HK, XM; mean age: 3;8, range: 3;0-4;4, two girls) who resided in rural villages of Debao county in Guangxi Province and were primarily cared for by their paternal grandmothers were recruited as the target group in this study (the GRA group). The grandmothers were in their fifties or sixties, with primary school education. All were native speakers of Zhuang with only basic knowledge of Mandarin. Another five children (ZL, XS, XT, LS, LC; mean age 3;4, range: 2;11-4;1, 3 girls) who were primarily cared for by their mothers in the urban areas of the same county were recruited as a comparison baseline group (the MOT group). The mothers had acquired Zhuang as their native language, but at the time of study, they were highly proficient and dominant in Mandarin. The MOT group had a higher socioeconomic status than the GRA group based on measures of education level (secondary/post-secondary vs. primary) and average monthly household income (CNY18,180 vs. CNY10,600, approximately USD2,500 and USD1,460 respectively), in addition to the geographical areas where the two groups of participants were recruited (urban vs. rural). Notice that although the two geographical areas are labelled as “urban” and “rural” here for ease of reference, they are only 10 kilometers apart and both are in the outskirts (rather than the centre) of Debao, located on the “rural” side of the urban-rural continuum. No language, hearing, brain or cognitive disorders of the children were reported by the caregivers. All children were ethnically Zhuang and were attending mainstream kindergartens with Mandarin as the medium of instruction.

The study adopted a Time 1/Time 2 design to compile language input profiles of the main caregivers at Time 1 and investigate to what extent input profiles compiled at Time 1 can account for bilingual outcomes at Time 2. The two data collection time points were two years and five months apart, roughly corresponding to the beginning and the end of the kindergarten period respectively. Caregivers were interviewed by a local bilingual assistant at two data collection time points to report their language use with the child on a five-point scale ranging from “almost exclusively in Zhuang” to “almost exclusively in Mandarin”. All grandmothers reported addressing the children “almost exclusively in Zhuang” at both time points, whereas the mothers reported using mostly Mandarin, with increasing proportions at Time 2: four of the five mothers spoke “mostly in Mandarin, sometimes in Zhuang” to their children at Time 1 and “almost exclusively in Mandarin” at Time 2 and one of them spoke “half Zhuang, half Mandarin” at Time 1 and “mostly in Mandarin, sometimes in Zhuang” at Time 2. Table 1 shows more details of the participants.

**Table 1:** Demographics of participants.

Child				Primary caregiver		
ID	Gender	Age (Time1)	Age (Time2)	Age (Time1)	Education	Language with child (Time1)
*Children cared for by Zhuang-grandmothers in rural villages (GRA group, n = 4)*						
**GRA**-JD	M	4;2	6;7	56	Primary	Almost exclusively in Zhuang
**GRA**-JR	F	3;0	5;5	58	Primary	Almost exclusively in Zhuang
**GRA**-HK	M	4;4	6;8	58	Primary	Almost exclusively in Zhuang
**GRA**-XM	F	3;4	5;9	66	Primary	Almost exclusively in Zhuang
*Children cared for by bilingual mothers in urban town (MOT group, n = 5)*						
**MOT**-ZL	F	3;5	5;10	41	Junior secondary	Half Zhuang, half Mandarin
**MOT**-XS	F	4;1	6;6	39	Junior secondary	Mostly Mandarin, sometimes Zhuang
**MOT**-XT	F	2;11	5;4	27	Junior secondary	Mostly Mandarin, sometimes Zhuang
**MOT**-LS	M	3;3	5;8	29	Associate degree	Mostly Mandarin, sometimes Zhuang
**MOT**-LC	M	3;3	5;8	33	Vocational secondary	Mostly Mandarin, sometimes Zhuang

### Tasks, materials and procedures

To collect language input data at Time 1, a Zhuang-Mandarin bilingual research assistant recorded interaction of individual caregiver-child dyads in a storytelling task in the way they normally interacted with each other in similar activities. Completion time of this task ranges from 5 to 10 minutes.

At Time 2, the children were tested for productive vocabulary in Mandarin and Zhuang using the Multilingual Naming Test (MiNT, [30]), and invited to complete narration tasks in Mandarin and Zhuang based on the Multilingual Assessment Instrument for Narratives (MAIN, [31, 32]). Time 2 testing was administered by research assistants via Zoom, with the company of a local assistant and the (grand)parents. MiNT and MAIN have been shown to be effective tasks in studies with ethnic minority children in rural China (e.g., [33]).

MiNT is a picture-naming task assessing productive vocabularies of multilingual children through a set of 68 black-and-white line drawings, ordered by estimated increasing difficulty, with one score assigned to each picture. Following the standard procedures of MiNT, semantic and phonetic cues were provided if the child was unable to provide the target response. The test ended after six consecutive failures. For MAIN, all children were required to tell the Cat and the Baby Birds stories in Mandarin and the Dog and the Baby Goats stories in Zhuang, as well as answer comprehension questions about individual stories (10 questions per story). In the absence of existing Zhuang adaptation of the grading rubrics for MiNT and MAIN, we compiled Zhuang versions of the materials in the following two steps: (i) three Zhuang-Mandarin bilingual research assistants trained by Guangxi Minzu University and the Chinese University of Hong Kong translated the instructions and prompts in the Mandarin version of MiNT and MAIN into Zhuang and generated the acceptable responses for both MiNT and the comprehension questions of MAIN, and (ii) a Zhuang-Mandarin bilingual researcher with doctoral training in linguistics originally from the same county as the participants checked the materials for grammaticality, accuracy and cultural appropriateness.

The testing typically lasted around 1.5 hours. The two Mandarin tests were completed in one session and the Zhuang ones in another, with a short break between sessions. The vocabulary test was administered before the narration task in the same language, and the order of languages counterbalanced across participants so that two GRA and two MOT children were randomly selected from the pool to complete the Zhuang tasks before the Mandarin ones. The Mandarin tasks were administered and assessed by research assistants who were native speakers of Mandarin with no knowledge of Zhuang, and the Zhuang tasks by a trained Zhuang-Mandarin research assistant. All participants completed the vocabulary tests in both languages and storytelling in Mandarin, whereas only those in the GRA group were able to tell the stories in Zhuang. The aims and methods of the study were reviewed and approved by the Survey and Behavioural Research Ethics Committee of Chinese University of Hong Kong. Recruitment and data collection for Time 1 started on 1 July 2021 and ended on 6 August 2021, and data collection for Time 2 started on 9 December 2023 and ended on 24 December 2023. Importantly, one of the authors (Liao) is a native of Debao county and scholar involved in language preservation regarding Zhuang. He was involved in this study from its conception to conclusion. The objectives and procedures of the study were explained to potential participants through this author and his local network, with the help of student assistants who were also natives of Guangxi. Participation was completely voluntary, and all personal and identifying data were kept confidential and removed from the record sheets before data analysis. The children were accompanied by a parent/guardian and a local assistant at all times, and were encouraged to take breaks between tasks during the testing at Time 2. Written informed consent from individual parents was obtained prior to data collection at Time 1.

### Transcription, data coding and measures of the speech samples

Caregiver-child interactions at Time 1 and child narratives at Time 2 were manually transcribed in the CHAT format in CLAN [34] by five trained research assistants, and then checked and revised for accuracy by the bilingual researcher. The morphological tiers of the Mandarin speech samples were automatically generated using the MOR function in CLAN. Due to the lack of a Zhuang lexicon in MOR, the assistants and the Zhuang researcher manually typed in the morphological tiers. Several measures were generated based on the morphological tiers of the transcripts (e.g., Total Utterance, Mean Length of Utterances in words/MLUw, Mean Length of the three longest Utterances/MLU3, word type, type/token ratio). For the narrative samples, children’s utterances that were not part of the storytelling (e.g., conversing with the experimenters) were excluded from the analysis.

For the narratives, we conducted additional analyses of the macro- and micro-structures. **Macrostructure** was evaluated by three measures adopted from Gagarina *et al*. [31] and Sheng *et al*. [35]: (i) Story Structure (SS) which measures the number of five types of components including setting, goals, attempts, outcomes, and internal state terms (max. 17 points), (ii) Story Complexity (SC) which considers the [Goal-Attempt-Outcome] ([GAO]) structures produced, including variants such as [AO], [G], [AG] and [GO]; only structures that include a goal statement [G] were awarded one point (max. 3 points); and (iii) comprehension questions evaluated comprehension of goals, internal state terms and theory of mind, with 1 point assigned to a question (max. 10 points). For **microstructure**, in addition to general measures such as MLU, MLU3 and Word Type, we also scrutinized 21 grammatical structures subsumed under four grammatical domains (i.e., phrase, modifier, nominal, verb). Selection of these structures was based on a comprehensive review of previous microstructure analyses [35–37]. Each utterance was manually coded by a trained research assistant, who calculated the raw frequency of each microstructure for each participant and obtained the group means. For macrostructure, a second coder coded all the transcripts independently (Cohen’s kappa = .772), then both coders examined all inconsistent results and revised their codings until a consensus was reached. The revised coding results are reported below. For microstructure, intercoder reliability was established by a second coder coding a random sample of 20% of the transcripts (Cohen’s kappa = .900).

## Results

### Grandmaternal and maternal input in Zhuang and Mandarin at Time 1

To understand fine-grained properties of the input provided by the grandmothers and the mothers, we analyzed the caregiver-child interaction recordings for total utterances, grammatical complexity (MLUw) and lexical diversity (type/token ratio, TTR) in the (grand)maternal input. Results are shown by measure and language in Table 2. The grandmothers used predominantly Zhuang to address the children, with the majority of the utterances purely in Zhuang (M = 84%), followed by a small proportion mixing Zhuang and Mandarin (M = 15%). The grandmothers almost never addressed the children in pure Mandarin sentences (M = 1%). In contrast, there was wide variation in the distribution of Zhuang, Mandarin and mixed utterances in the maternal input, ranging from almost exclusively pure Mandarin (XS, XT, LS), and close-to-balanced use of pure Zhuang, pure Mandarin and mixed utterances (LC), to Zhuang-dominant (ZL). In terms of quality of the input, grandmaternal input in Zhuang was within the range of maternal input in both languages in terms of grammatical complexity (${\text{MLUw}}_{\text{GRA}}=4.37$-6.28, ${\text{MLUw}}_{\text{MOT}}=4.05$-6.39), but below the range of the maternal input in lexical diversity (${\text{TTR}}_{\text{GRA}}=\mathrm{.15}$-.20, ${\text{TTR}}_{\text{MOT}}=\mathrm{.17}$-.45). Overall, the results suggest that the grandmothers from the rural villages provided large amounts of input in Zhuang to the GRA children in toddlerhood, whereas the mothers in the urban town areas used considerably less Zhuang with their children, if any. The Zhuang input provided by the grandmothers was similar to the language input provided by the mothers in terms of grammatical complexity, albeit lower in lexical diversity.

**Table 2:** Characteristics of input provided by (grand)mothers at Time 1.

Child ID	Total	Zhuang			Mandarin			Mixed
		Uttr (%)	MLUw	TTR	Uttr (%)	MLUw	TTR	Uttr (%)
*Grandmothers in rural villages (GRA group, n = 4)*								
GRA-JD	136	133 (98%)	6.28	.20	0 (0%)	–	–	3 (2%)
GRA-JR	338	258 (76%)	5.33	.15	2 (1%)	–	–	78 (23%)
GRA-HK	190	154 (81%)	5.05	.15	1 (1%)	–	–	35 (18%)
GRA-XM	240	218 (91%)	4.37	.19	3 (1%)	–	–	19 (8%)
*Mean*	*226*	*190.75 (84%)*	*5.26*	*.17*	*1.5 (1%)*	–	–	*33.75 (15%)*
*Mothers in urban town areas (MOT group, n = 5)*								
MOT-ZL	118	92 (78%)	6.39	.25	4 (3%)	–	–	22 (19%)
MOT-XS	184	0 (0%)	–	–	184 (100%)	5.43	.27	0 (0%)
MOT-XT	183	1 (1%)	–	–	180 (98%)	5.15	.17	2 (1%)
MOT-LS	251	1 (1%)	–	–	250 (99%)	5.00	.19	0 (0%)
MOT-LC	245	95 (39%)	5.17	.19	55 (22%)	4.05	.45	95 (39%)
*Mean*	*196.2*	*37.8 (19%)*	*5.78*	*.22*	*134.6 (69%)*	*4.91*	*.27*	*23.8 (12%)*

Notes: MLUw = Mean Length of Utterance in words, TTR = Type/token ratio; for cases with fewer than 5 utterances, MLUw and TTR were not calculated.

### Bilingual development at Time 2

Table 3 reports the children’s performance in the vocabulary test (MiNT) and the narration task (two picture stories per language) in Zhuang and Mandarin. For the purpose of between-group comparison, we calculated the “standardized ranges” of the measures in the MOT children by identifying the range between one standard deviation (SD) above the mean and one SD below the mean (mean ± 1SD) for each performance measure.

**Table 3:** Child outcomes at Time 2 (vocabulary tests and narratives).

Child	Language	Vocab	Elicited narration (per story)						
		Score	Ttl uttr	Macrostructure			Microstructure		
				SS	SC	CS	MLUw	MLU3	Type
GRA-JD	Zhuang	19	4.00	*–*	*–*	*2.50*	*–*	*–*	*–*
GRA-JR	Zhuang	33	0.50	–	–	2.00	–	–	–
GRA-HK	Zhuang	44	6.00	–	–	6.00	–	–	–
GRA-XM	Zhuang	3	3.50	–	–	3.50	–	–	–
***GRA-Mean***	***Zhuang***	***24.75***	***3.50***	*–*	*–*	***3.50***	*–*	*–*	*–*
GRA-JD	Mandarin	45	14.00	6.00	1.00	8.00	6.07*	8.67*	38.00
GRA-JR	Mandarin	52	19.50	8.00	1.00	7.00*	7.60	13.17	55.00
GRA-HK	Mandarin	49	11.50	6.50	2.00	7.50*	6.90	11.33	33.00
GRA-XM	Mandarin	47	13.00	8.00	2.00	7.00*	6.32	9.33	44.00
***GRA-Mean***	***Mandarin***	***48.25***	***14.50***	***7.13***	***1.5***	***7.38***	***6.72***	***10.63***	***42.50***
MOT-ZL	Zhuang	3	*–*	–	–	–	–	–	–
MOT-XS	Zhuang	2	*–*	–	–	–	–	–	–
MOT-XT	Zhuang	0	*–*	–	–	–	–	–	–
MOT-LS	Zhuang	0	*–*	–	–	–	–	–	–
MOT-LC	Zhuang	2	*–*	–	–	–	–	–	–
***MOT-Mean***	***Zhuang***	***1.40***	*–*	*–*	*–*	*–*	*–*	*–*	*–*
MOT-ZL	Mandarin	53	30.00	10.50	0.50	8.50	8.75	15.50	87.00
MOT-XS	Mandarin	57	10.00	6.00	0.50	8.50	9.35	12.83	45.00
MOT-XT	Mandarin	38	15.50	7.00	0	7.50	8.09	13.00	53.50
MOT-LS	Mandarin	42	14.00	7.50	0.50	7.50	7.52	12.17	42.00
MOT-LC	Mandarin	47	9.50	6.50	2.00	9.00	5.36	6.50	27.00
***MOT-Mean***	***Mandarin***	***47.40***	***15.80***	***7.50***	***0.70***	***8.20***	***7.81***	***12.00***	***50.90***
***+1SD***	***Mandarin***	***55.17***	***24.14***	***9.27***	***1.46***	***8.87***	***9.35***	***15.32***	***73.23***
** −1SD **	***Mandarin***	***39.63***	***7.46***	***5.73***	***–0.06***	***7.53***	***6.28***	***8.68***	***28.57***

Notes: Data for the narration task show the averages of the two picture stories; Ttl uttr = total utterances in the narratives, SS = Story Structure, SC = Story Complexity, CS = Comprehension Score, MLUw = Mean Length of Utterance in words, MLU3 = Mean Length of three longest Utterances, Type = word type; * = below the standardized ranges of the MOT children; some data were missing due to lack of sufficient analyzable utterances.

All children completed the vocabulary test in both languages. For Mandarin, the GRA children displayed a slightly higher mean than the MOT children (${\text{M}}_{\text{GRA}}=48.25$, ${\text{M}}_{\text{MOT}}=47.40$), and their individual scores fell within the range of the MOT children, whether judged by the range of the raw scores or the standardized ranges of the MOT children. For Zhuang, the GRA children showed an obvious advantage over the MOT children, as three out of four children (JD, JR, HK) obtained high scores in this productive vocabulary task (19-44), whereas none of the five MOT children was able to name more than three pictures correctly in the task (M = 1.40). All children, regardless of grouping, achieved higher scores in Mandarin than in Zhuang, suggesting stronger productive vocabulary in Mandarin at this stage.

Similarly, results of the narration task revealed between-group and between-language differences. Both groups were able to tell stories in Mandarin, and the lengths of the stories (total utterance per story) by the GRA children fell within the raw and standardized ranges of the MOT children. However, only the GRA children were able to provide narratives in Zhuang and answer the comprehension questions in Zhuang. Their narratives in Zhuang consisted of only a few utterances per story (Mean ${\text{Utterance}}_{\text{GRA}}=3.50$) and were thus much shorter than those in Mandarin. Despite multiple prompts by the research assistants, none of the MOT children was able to tell the stories or answer the comprehension questions in Zhuang. Given the lack of analyzable utterances in Zhuang, we did not compute macro- or micro-structure measures for this language, except for the comprehension scores in the GRA children (M = 3.50), which were lower than the corresponding scores in Mandarin across individuals (M = 7.38). Focusing on macrostructure in the Mandarin narratives, individual GRA children performed within the standardized ranges of the MOT children across measures, except for comprehension score. In terms of micro-structures, only one out of four children (JD) fell below the standardized ranges of the MOT children in two of the three microstructure measures. In sum, the results in this section revealed superior performance in Zhuang by the GRA children and slightly lower performance in Mandarin in the GRA children compared with the MOT children in the vocabulary and narration tasks at Time 2.

### Grammatical development in Mandarin in the Zhuang-Mandarin bilinguals

As described in the methods section, we coded 21 grammatical structures in individual narratives to assess grammatical development in the children. Given the lack of analyzable narratives in Zhuang, we coded Mandarin narratives only. Table 4 reports the raw counts of the structures produced by individual children in Mandarin. Structures produced at least twice by the child were considered to have emerged in the child’s Mandarin production and are indicated by shaded cells. The results revealed a number of similarities between the children from the GRA and MOT groups: i) classifiers, personal pronouns and perfective markers, which have close structural equivalents in Zhuang and should be highly frequent in daily language use, had emerged and were quite productive across individual children, ii) relative clauses, quantifiers and verb reduplication were less frequently produced and likely late acquired, and iii) the other structures manifested large individual variation among the children.

**Table 4:** Grammatical structures in the elicited narratives of the bilingual children (coding criterion following [37]).

(l0ptr3pt)1-13		GRA group (n = 4)					MOT group (n = 5)					
		JD	JR	HK	XM	Mean	ZL	XS	XT	LS	LC	Mean
**Phrase**	“BA”	0	3	3	5	***2.75***	2	0	2	1	0	***1.00***
	“BEI”	1	3	0	2	***1.50***	9	1	0	0	0	***2.00***
	Locative	6	3	3	3	***3.75***	10	5	11	2	1	***5.80***
	Temporal	0	5	1	2	***2.00***	1	3	0	0	0	***0.80***
	Relative clause	0	1	1	0	***0.50***	2	1	3	0	0	***1.20***
**Modifier**	Adjective	23	10	18	20	***17.75***	29	11	22	5	0	***13.40***
	Adverb	4	22	0	8	***8.50***	10	2	6	7	2	***5.40***
	Negation	7	9	7	3	***6.50***	7	5	1	9	7	***5.80***
	Quantifier	0	1	0	0	***0.25***	5	0	0	2	0	***1.40***
**Nominal**	Classifier	6	25	4	3	***9.50***	52	4	10	26	2	***18.80***
	Possessive	1	6	2	2	***2.75***	6	4	4	3	0	***3.40***
	Demonstrative	1	8	1	0	***2.50***	24	0	3	14	0	***8.20***
	Personal pron.	3	26	3	2	***8.50***	17	3	3	12	2	***7.40***
**Verb**	Progressive asp.	2	8	4	2	***4.00***	5	10	1	0	0	***3.20***
	Perfective asp.	19	20	4	17	***15.00***	20	15	15	18	12	***16.00***
	Reduplication	0	0	0	0	***0***	0	0	0	0	0	***0***
	Resultative	0	2	1	1	***1.00***	4	0	0	3	3	***2.00***

Notes: “BA” typically appears in [S-*ba*-O-VP] and is subject to thematic and event semantic constraints; “BEI” is a passive construction and usually appears in [O-*bei*-S-VP]; shaded cells represent structures produced at least twice by the child.

## Discussion

### Summary of findings

This study was motivated by a long-standing gap in research on linguistic properties of language input provided by grandparents in heritage and majority language development, as well as the unique language profile of the Zhuang-Mandarin bilingual populations in Guangxi. Our research questions comparing the bilingual input and outcomes between two groups of children (GRA, MOT) guided our study and analysis. Through a combination of (grand)parental questionnaire, caregiver-child interaction recording, picture naming and elicited narration tasks, we collected data in two waves when the children had just started Mandarin-medium kindergarten (mean age 3;6) and were approaching the end of it (mean age 5;11) two years and five months later, focusing on the input in the first wave and the outcomes in the second.

Our analysis of caregiver-child recording at Time 1 shows that the GRA children from rural villages had heard large amounts of input in Zhuang (84%), some Zhuang-Mandarin mixed input (15%) and almost no pure Mandarin utterances from their grandmothers, whereas their MOT peers in the urban town areas heard a larger proportion of Mandarin (69%) and a smaller proportion of Zhuang (19%) from their mothers. Additionally, the Zhuang input provided by the grandmothers is similar to the maternal input in grammatical complexity, but does not match the maternal input in lexical diversity.

At Time 2, the children’s performance revealed considerable between-group and between-language differences. All children, whether GRA or MOT, performed significantly better in Mandarin than in Zhuang, whether in productive vocabulary or narrative skills. The MOT children obtained extremely low scores in Zhuang vocabulary (M = 1.40 out of 68) and were unable to produce narratives in Zhuang at all. The GRA children, on the other hand, scored much higher than the MOT children in Zhuang vocabulary (M = 24.75), and were able to produce stories in Zhuang and answer some standardized comprehension questions about the stories in Zhuang, although their storeis were quite short (M of utterance = 3.50) and the scores of their answers were low (M = 3.50) compared to their performance in the Mandarin counterparts. In terms of narrative skills in Mandarin, the means of macro- and micro-structure measures were slightly lower in the GRA children than the MOT children, but individual analysis shows that few GRA children fell below the “standardized ranges” of the MOT children. Our analysis of 21 grammatical structures in Mandarin elicited through the narration task revealed a similar productivity ranking between the GRA and MOT children, with productive use of classifiers, aspect markers and personal pronouns and little or no use of relative clauses, quantifiers and reduplication structures.

### Home language input and early development of Zhuang

While our study has a very small sample size (n = 9), our findings suggest that Zhuang, as an ethnic minority language, is being passed on to young Zhuang children primarily through grandparent-child interactions, rather than mother-child interactions, if at all. This is consistent with our prediction based on recent reports of the linguistic profiles of adult Zhuang speakers in Guangxi. What is remarkable is that the five mothers in this study were native speakers of Zhuang, and yet only one of them (mother of ZL) reported speaking Zhuang regularly to the child and was observed using a substantial amount of Zhuang to address the child in the caregiver-child interactions. All of the mothers had ceased speaking Zhuang with their children by the second time point when the children were completing Mandarin-medium kindergarten. Note that these mothers were better educated than the grandparents and came from households with higher socioeconomic status. Their choice of Mandarin over Zhuang could have various linguistic and non-linguistic motivations, such as Mandarin becoming the lingua franca in the larger community as a result of migration and urbanization, Mandarin being perceived as the prestigious language in school education and language policies, parental recognition of bilingual advantages and globalization (see [38] for parents’ ideologies concerning Mandarin-English bilingual education in urban China), among others. It is beyond the scope of this acquisition study to further discuss the motivations behind the mothers’ language choices since we did not collect data in this respect. Additionally, it is possible that mothers from a different socioeconomic class may have different language choices. Our findings have provided the first observational evidence that Mandarin, the societal majority language, has replaced Zhuang as the main language between mothers and children of Zhuang ethnicity in mid-SES urban households in Guangxi. Larger-scale research is needed to look into this on-going language shift from linguistic, educational and socioeconomic perspectives.

According to the measures of language vitality and endangerment by the United Nations Educational, Scientific and Cultural Organization [39], whether children are still learning the language is the first and foremost measure in determining language vitality. The vitality and sustainability of Zhuang hinges on whether or not it is acquired as a first language (most likely) by bilingual children along with Mandarin, and used by them across life domains. Intergenerational transmission of Zhuang from bilingual mothers to children will only happen when dual language input in both Zhuang and Mandarin is provided, that is, children have regular exposure to both Zhuang and Mandarin from their mothers. If mothers only speak Mandarin to their children, the children will not receive input in Zhuang and are expected to develop like monolingual children with some passive competence in Zhuang if they overhear it spoken in the family at all, as is often the case with heritage languages.

An encouraging finding is that the Zhuang input provided by the grandparents supported development of Zhuang in the children, as evidenced by the better performance in Zhuang in the GRA children compared with the MOT children at Time 2. Note that the grandmothers included in this analysis were from the rural villages with no more than primary school education. Given the well-established positive relation between caregivers’ education level and input quality, it is neither reasonable nor realistic to expect that the grandmaternal input will match the maternal input in terms of quality. Our data confirmed that the grandmothers’ input was lexically less diverse than the mothers’. This may partially explain why the children in the GRA group did not develop balanced oral proficiency in Zhuang and Mandarin at kindergarten age in spite of early and consistent grandmaternal input in Zhuang in toddlerhood. It remains to be seen whether grandparental input provided by higher-SES speakers (like those in Li & Matthews’ case study [2]) will differ from maternal input in the minority or majority language in terms of quality.

That being the case, the loss of Zhuang in the GRA children was alarmingly fast. Shift of language dominance from the minority L1 to the majority L2 accompanied by protracted development in the L1 is common among immigrant children in English-dominant societies [40]. The rapid shift from Zhuang to Mandarin during kindergarten age is, however, somewhat unexpected when one takes into consideration the status of Zhuang as a “major” minority language in the polity. Zhuang was not recently brought into the region and spoken by only scattered individuals or communities. Rather, it is a language native to Guangxi, spoken by 20 million speakers and receiving official recognition and protection by the central and provincial governments through various forms of bilingual education and Zhuang-inclusive media [41]. This is a fundamental difference between the Zhuang-Mandarin children under investigation and the second-generation immigrant children extensively studied in the heritage language acquisition literature in terms of linguistic landscape. Young children who are ethnically Zhuang and cared for by Zhuang-native caregivers in this Zhuang-speaking community should have easy and regular access to at least spoken Zhuang and should develop at least fluent oral skills. The loss of Zhuang by the end of kindergarten in those children underscores the importance of formal education and literacy in the maintenance of the heritage language in addition to early and rich oral input. This is gist of “The Literacy Enhancement Hypothesis” [42], an emerging research theme in heritage language acquisition.

### Catching up in Mandarin: a comparison with Sheng *et al*. [35]

Given the similarities between Zhuang and Mandarin grammars, we predicted that the GRA children might benefit from positive transfer from Zhuang to Mandarin and perform similarly with their MOT peers at Time 2. The vocabulary score and multiple measures derived from the elicited narratives largely confirmed this prediction. Note that both the GRA and MOT children showed the most productive use of classifiers, personal pronouns and perfective aspect markers, which, as reviewed above, are grammatical elements shared by Zhuang and Mandarin. This shows that in the presence of high levels of similarity between languages, reinforcement and acceleration effects can appear at very early stages of bilingual exposure, mitigating potential negative effects of reduction of input in specific languages (see also [3]). Our findings provide new evidence of majority language development from a previously unstudied language pair, in which both are isolating languages. As a limitation, our study elicited mainly production performance and did not examine comprehension and perception of specific linguistic details, and the measures we used only captured outward linguistic behaviours rather than underlying psychological or neural mechanisms. However, both vocabulary size and narrative skills have served as valid assessment tools widely used across bilingual and monolingual, typical and atypical populations and are among the strongest predictors for academic achievements during school age [35, 37, 43]. For children, parents and educators, these are indicators of upmost importance.

Notes: The -1SD statistics were calculated by deducting one SD value from the mean of the TD group; * = below the standard range of the Nanjing children.

Our study did not include a control group of monolingual children because our primary goal was to compare children receiving input from grandmothers and mothers respectively. In this discussion, in order to place our findings against the broader context of child Mandarin, we draw from the results reported in Sheng *et al*. [35], who tested 21 typically developing monolingual Mandarin children (4;2-6;8, mean age 5;7) in Nanjing, the capital city of Jiangsu Province, among others, using the same MAIN tasks as in our studies. In Table 5 below, we juxtapose two common macro- and micro-structure measures in the two studies for easy comparison. The three groups of children were quite similar in the MLUw, but there is a clear gap between the Guangxi and Nanjing children in terms of story structure, whether judged by the group means or the standardized range calculated based on the Nanjing sample. It is unclear to what extent the performance gap can be attributed to the bilingual versus monolingual experience or other important differences between the two samples. For instance, the Guangxi and Nanjing children differ in their SES as indexed by the maternal education level – mothers of the Nanjing children were better educated with an average education between post-secondary diploma and university degree, ranging from secondary school to master’s degree. Another advantage of the Nanjing children is that they were administered with both storytelling and retelling tasks in the original study, with the retelling task administered before the telling task. Although the results in Table 5 were pulled from the telling task, the provision of a model story in the retelling part may have elevated their performance in the subsequent telling task for the Nanjing children. Future studies may follow up on this and examine different attainment in macrostructure in storytelling tasks in Zhuang-Mandarin and Mandarin monolingual children matched for SES and relevant variables and effective interventions to boost their performance.

**Table 5:** Comparing narrative measures of our study and Sheng *et al*. [35].

	This study		Sheng *et al*. [35]
	GRA group	MOT group	TD group (n = 21)
**Location**	Debao, Guangxi		Nanjing, Jiangsu
**Mean age**	5;11	6;1	5;7
**Macrostructure (Story Structure score)**	7.13*	7.50*	9.52 (-1SD = 7.77)
**Microstructure (MLUw)**	6.72	7.81	7.38 (-1SD = 6.29)

Notes: The -1SD statistics were calculated by deducting one SD value from the mean of the TD group; * = below the standard range of the Nanjing children.

### Sequential bilingualism in Zhuang and Mandarin: implications for language planning and intervention

For many children growing up in rural areas of China where a minority language (or a regional dialect) is used at home and the majority language in the school system, sequential bilingualism and shift of language dominance is a stark reality that is largely inevitable, and yet there are important advantages associated with early exposure to the minority language that have not been fully recognized or put into use in order to preserve the minority language and its cultural legacy. Numerous studies have compared heritage language learners (even those who are only passive overhearers), who turn to the classroom to re-learn or expand their knowledge in their heritage L1 with adult L2 learners, who spend an equal amount of time with the heritage language learners learning the language or demonstrate equal general proficiency in the language. They found that heritage language learners usually outperform those true post-puberty L2 learners in tasks that tap into implicit (rather than explicit) knowledge of the language, reflecting the modality and setting in which they acquire their L1 (see [12, 44] for reviews). This advantage of heritage language speakers has been widely documented across languages and linguistic domains, from the most basic phonological properties (e.g., [45, 46]) to highly complex morphosyntactic structures and discourse features known to cause persistent difficulties for adult L2 learners (e.g., [47, 48]). These findings unanimously show that substantial exposure to the heritage language in early childhood makes it easier for the learners to brush up on the language later in adolescence and adulthood, despite pauses and reduction of input and experience in between. If additive bilingualism of Zhuang and Mandarin is the long-term educational goal, early exposure to Zhuang through naturalistic interactions with caregivers should be actively and directly promoted in both rural and urban households. Early language intervention programmes can consider compiling additional resources and providing practical suggestions for rural households where the grandparents are the main source of Zhuang input in order to improve the quality of input and exposure of Zhuang-learning children in the rural areas. Given early exposure plus continued educational support, it is hoped that those early sequential bilinguals will develop into proficient and confident speakers of Zhuang, who will pass on Zhuang as a living language to the next generation.

On the other side of the coin, our comparison of Mandarin proficiency among three groups of Mandarin-learning children (GRA and MOT children in our study; monolingual TD children in [35]) has identified specific gaps between the ethnically Zhuang children and monolingual Mandarin children in terms of Mandarin skills. Although it is not clear whether the gaps were caused by bilingualism, SES, data elicitation mode, or some combination of these factors, the magnitude of the gaps is smaller than one would expect for early bilinguals in general, probably due to long-term contact between the languages and systematic similarities between Zhuang and Mandarin across phonological, lexical and grammatical levels. To our knowledge, however, the interdependent nature of the two languages in the developing bilingual and the systematic cross-linguistic correspondence has not been recognized as a linguistic asset of the Zhuang-speaking learners and effectively incorporated in Zhuang-Mandarin bilingual curriculum or language assessment [9, 41, 49]. As a reviewer points out, exploring translanguaging strategies in language pairs with high structural similarity could significantly advance the research on practical approaches to heritage language maintenance. With Zhuang being a “major” minority language relatively close to Mandarin in both language distance and prestige, the Zhuang-Mandarin bilingual classroom is an ideal testing ground for effectiveness of translanguaging as a pedagogy for both teaching and learning, a frontier in bilingual education research [50, 51].

## Conclusions

This study has a limitation of a very small sample (n = 9), and the language proficiency measures employed mostly tapped productive, rather than receptive knowledge, and may have failed to capture signs of development, maintenance or attrition in language comprehension and processing. Future studies should consider replicating the findings with a larger sample from a wider yet more controlled range of socioeconomic backgrounds using a comprehensive set of language measures. Nevertheless, this study is the first to document an on-going language shift from Zhuang to Mandarin in ethnically Zhuang households in rural and urban Guangxi through the lens of bilingual development and caregiver-child interactions in kindergarteners. Our findings show that grandparents are effective providers of language input in Zhuang, which supports the children’s development in Zhuang until the children enter Mandarin-medium kindergarten. No evidence suggests that grandparental input in the minority language in toddlerhood has a negative impact on the development of the majority language at the kindergarten age. Given the findings, it is recommended that early sequential bilingualism taking advantage of linguistic correspondences between Zhuang and Mandarin as well as actively involving the grandparents as main or additional providers of Zhuang input in the family is beneficial to both preservation of Zhuang as a minority language and mastery of the national majority language. With resources and support from the society and the family, the caring Chinese grandmothers have the power to turn the tide and reverse the loss of a heritage language, whether in the US [2] or rural China.
